# Efficient federated learning for distributed neuroimaging data

**DOI:** 10.3389/fninf.2024.1430987

**Published:** 2024-09-09

**Authors:** Bishal Thapaliya, Riyasat Ohib, Eloy Geenjaar, Jingyu Liu, Vince Calhoun, Sergey M. Plis

**Affiliations:** ^1^Translational Research In Neuroimaging and Data Science Center, Atlanta, GA, United States; ^2^Department of Computer Science, Georgia State University, Atlanta, GA, United States; ^3^School of Electrical and Computer Engineering, Georgia Institute of Technology, Atlanta, GA, United States

**Keywords:** efficient federated learning, neuroimaging, sparse models, communication efficiency, sparsity

## Abstract

Recent advancements in neuroimaging have led to greater data sharing among the scientific community. However, institutions frequently maintain control over their data, citing concerns related to research culture, privacy, and accountability. This creates a demand for innovative tools capable of analyzing amalgamated datasets without the need to transfer actual data between entities. To address this challenge, we propose a decentralized sparse federated learning (FL) strategy. This approach emphasizes local training of sparse models to facilitate efficient communication within such frameworks. By capitalizing on model sparsity and selectively sharing parameters between client sites during the training phase, our method significantly lowers communication overheads. This advantage becomes increasingly pronounced when dealing with larger models and accommodating the diverse resource capabilities of various sites. We demonstrate the effectiveness of our approach through the application to the Adolescent Brain Cognitive Development (ABCD) dataset.

## 1 Introduction

Deep learning has transformed fields like computer vision, natural language processing, and is also starting to transform the field of neuroimaging. As deep learning models grow, distributed and collaborative training becomes essential, especially when sensitive data is spread across distant sites. Collaborative MRI data analysis offers profound insights, allowing researchers to utilize data beyond a study's original scope. As MRI scans are often preserved, vast amounts of data accumulate across decentralized research sites. Training models on more data, while preserving data privacy is thus crucial. Aggregating data from different sources to a central server for training can however expose this sensitive information, raising ethical concerns. Federated Learning (FL), an emerging paradigm in machine learning aims to leverage this distributed data while maintaining privacy. It achieves this by enabling devices or organizations to train models locally and share only aggregated training updates instead of raw data.

In FL, a central server coordinates training, and client sites communicate only model parameters, keeping local data private. In the decentralized setting, the server usually doesn't exist and clients train a model collaboratively among themselves. However, challenges arise due to data's statistical heterogeneity, limited communication bandwidth, and computational costs. Various methods have been proposed to address the high communication and computational costs of federated learning. Inspired by the findings from the lottery ticket hypothesis (Frankle and Carbin, 2019) which discovered that there exists *sub-networks* (a subset of network parameters within the larger complete neural network) which can be trained in isolation to almost full accuracy, many methods were proposed to train and update only a sub-network in the client sites (Dai et al., 2022; Huang H. et al., 2022). However, finding these sub-networks in the traditional method (Frankle and Carbin, 2019) is extremely computationally intensive and thus FL methods that rely on them (Huang H. et al., 2022) also share the same issues. Although initiating the federated training process from a random sub-network and updating the network in later work (Dai et al., 2022) brought about the benefits of both computational and communication efficiency, it came at the cost of performance due to starting the FL training process with random sub-networks. In this work we aim to solve this issue of starting from random sub-networks for the sparse FL process, targeted toward neuroimaging data.

We introduce *Sparse Federated Learning for NeuroImaging* or NeuroSFL a communication efficient federated learning method that identifies salient sub-networks at each client sites and trains sparse local models, greatly reducing the communication bandwidth. A notable difference of our method in contrast to competiting methods such as DisPFL (Dai et al., 2022) is that, NeuroSFL enjoys the benefits of sparse models at local cites such as faster inference (Dey et al., 2019) on top of the communication efficiency of sparse communications methods (Vahidian et al., 2021; Dai et al., 2022; Isik et al., 2022).

### 1.1 Contributions

NeuroSFL is a sparse federated learning method that discovers a common sub-network from the available data distributed across local sites and trains sparse local models leveraging the distributed data. Our key contributions are as follows:

1. We introduce NeuroSFL, a communication efficient federated learning approach geared toward training on distributed neuroimaging data in different client sites.
2. Our method identifies a global common sub-network at initialization and keeps this sub-network static throughout the federated learning process. Consequently, it only needs to share the sub-network masks *only once* before training begins, and never again, significantly reducing the communication overhead during training.
3. NeuroSFL does not need to share dense model parameters or masks during the training phase as it starts with a common initialization and only transmit sparse parameters each communication round depending on the chosen sparsity level.
4. We validate our method in a neuroimaging task and demonstrate its efficacy compared to competing methods.
5. Finally, unlike most competiting methods, to test the effectiveness of NeuroSFL, we also deploy and evaluate it in a real-world federated learning framework called COINSTAC (Plis et al., 2016) that trains neuroimaging models and report wall-clock time speed up.

## 2 Backgrounds and related works

In this section, we provide the necessary background for this work by introducing the federated learning problem in Section 2.1. We then discuss the related works in Section 2.3.

### 2.1 Federated learning

Federated Learning (FL) (McMahan et al., 2017) represents a novel approach in machine learning, facilitating model training across numerous decentralized devices or servers that hold local data samples without needing to exchange them. This contrasts sharply with traditional distributed learning methods, which centralize data and distribute computations. FL prioritizes privacy preservation, efficient communication, and resilience in diverse, heterogeneous environments. It diverges from conventional distributed learning paradigms, due to its distinct characteristics, some of which we detail below:

1. Non-IID data: the training data across different clients are not identically distributed, which means that the data at each local site may not accurately represent the overall population distribution.
2. Unbalanced data: the amount of data varies significantly across clients, leading to imbalances in data representation.
3. Massive distribution: often, the number of clients exceeds the average number of samples per client, illustrating the scale of distribution.
4. Limited communication: communication is infrequent, either among clients in a decentralized setting or between clients and the server in a centralized setting, due to slow and expensive connections.
5. Heterogeneous devices: clients in FL may have diverse computational capabilities, ranging from powerful servers to resource-constrained mobile devices.
6. Privacy preservation: FL is designed to ensure that raw data never leaves the clients' devices, preserving user privacy. Instead of sharing data, only model updates are shared. Although more sophisticated techniques have been proposed to both break the privacy guaranteed by vanilla FL (Geiping et al., 2020) and preserve the privacy (Zhang et al., 2023).
7. Local training: each client performs local training on its own data and only shares updates (e.g., weights or gradients) with the central server, which then aggregates these updates to improve the global model.
8. Client availability: clients may be intermittently available due to power constraints, connectivity issues, or user activities, requiring the system to be robust to varying participation.
9. Scalability: FL frameworks are designed to handle a large number of clients, scaling from hundreds to potentially millions of devices.

One of the main focuses of this work is to reduce the communication costs between the server and clients in a centralized setting or among clients in a decentralized setting when dealing with non-IID and unbalanced data. This is achieved by identifying a sub-network based on the data distributions at each local site and transmitting only the parameters of this sub-network in each communication round *r*. In each round, a fixed set of $\tilde{K}$ clients is sampled from all *K* clients, and federated training continues on the selected sub-network of those clients. The general federated optimization problem encountered is detailed next.

### 2.2 Federated optimization problem

In the general federated learning (FL) setting, a central server tries to find a global statistical model by periodically communicating with a set of clients. The federated averaging algorithm proposed by Konečnỳ et al. (2016), McMahan et al. (2017), and Bonawitz et al. (2019) is applicable to any finite sum objective of the form


(1) $$\begin{array}{l} \underset{\theta \in {\mathbb{R}}^{d}}{\min}f\left(\theta \right),\;\text{where }f\left(w\right)=\frac{1}{n}\overset{n}{\underset{i=1}{\sum }}f_{i}\left(\theta \right). \end{array}$$

In a typical machine learning problem, the objective function *f*_*i*_(*θ*) = *ℓ*(*x*_*i*_, *y*_*i*_; θ) is encountered, where the *i*^th^ term in the sum is the loss of the network prediction on a sample (*x*_*i*_, *y*_*i*_) made by a model with parameter θ. We assume that the data is partitioned over a total of *K* clients, with $P_{k}$ denoting the set of indices of the samples on client *k*, and $n_{k}=|P_{k}|$. The total number of samples *n* is given by $n=\overset{K}{\underset{k=1}{\sum }}n_{k}$. Thus, the objective in Equation 1 can be re-written as follows in Equation 2


(2) $$\begin{array}{l} f\left(\theta \right)=\overset{K}{\underset{k=1}{\sum }}\frac{n_{k}}{n}F_{k}\left(\theta \right),\text{where}F_{k}\left(\theta \right)=\frac{1}{n_{k}}\underset{i\in P_{k}}{\sum }f_{i}\left(\theta \right). \end{array}$$

In the typical distributed optimization setting, the IID assumption is made, which says the following: if the partition $P_{k}$ was created by distributing the training data over the set of clients uniformly at random, then we would have $E_{P_{k}}\left[F_{k}\left(\theta \right)\right]=f\left(\theta \right)$, where the expectation is over the set of examples assigned to a fixed client *k*. In this work, we consider the non-IID setting where this does not hold and *F*_*k*_ could be an arbitrarily bad approximation to *f*.

When designing an FL training paradigm, a set of core considerations have to be made to maintain data privacy and address *statistical* or *objective* heterogeneity due to the differences in client data and resource constraints at the client sites. A range of work tries to address the issue of heterogeneous non-IID data (McMahan et al., 2016; Kulkarni et al., 2020), however, some research also suggests that deterioration in accuracy in the FL non-IID setting is almost inevitable (Zhao et al., 2018).

### 2.3 Related works

In this section, we discuss the relevant literature in relation to NeuroSFL. First, in Section 2.3.1, we describe the role of federated learning in neuroimaging and discuss the relevant literature. Second, in Section 2.3.2, we introduce key works on model pruning and sparsity in deep learning, findings from which we leverage for formulating NeuroSFL. Third, in Section 2.3.3, we describe applications of model pruning and sparsity in the FL setting for efficient FL. Finally, in Section 2.3.4, we briefly discuss privacy in the FL setting.

#### 2.3.1 Federated learning in neuroimaging

Over the past decade, the field of neuroimaging has strongly embraced data sharing, open-source software, and collaboration across multiple sites. This shift is largely driven by the need to offset the high costs and time demands associated with neuroimaging data collection (Landis et al., 2016; Rootes-Murdy et al., 2022). By pooling data from different sources, researchers can explore findings that extend beyond the initial scope of individual studies (Poldrack et al., 2013). The practice of sharing data enhances the robustness of research through larger sample sizes and the replication of results, offering significant benefits for neuroimaging studies. Even though data pooling and sharing data is embraced, there are significant challenges related to data privacy, security, and governance that limit the extent to which data can be shared. This is where FL becomes crucial as it enables collaborative model training across multiple institutions without the need to directly share sensitive data. Moreover, with FL collaborative training, sample size also plays a crucial role, where increasing the sample size not only makes predictions more reliable but also ensures the reliability and validity of research findings, thereby preventing data manipulation and fabrication (Tenopir et al., 2011; Ming et al., 2017). Furthermore, aggregating data can lead to a more diverse sample by combining otherwise similar datasets, thus reflecting a broader range of social health determinants for more comprehensive results (Laird, 2021). Additionally, reusing data can significantly reduce research costs (Milham et al., 2018).

FL is increasingly recognized as a transformative approach in healthcare and neuroimaging. In the realm of biomedical imaging, FL has been applied to a variety of tasks. These include whole-brain segmentation from MRI T1 scans (Roy et al., 2019), segmentation of brain tumors (Li et al., 2019; Sheller et al., 2019), multi-site fMRI classification, and the identification of disease biomarkers (Li X. et al., 2020). COINSTAC (Plis et al., 2016) offers a privacy-focused distributed data processing framework specifically designed for brain imaging showcasing FL's role in enhancing privacy and efficiency in healthcare data analysis. Additionally, it has been utilized in discovering brain structural relationships across various diseases and clinical cohorts through federated dimensionality reduction from shape features (Silva et al., 2019).

#### 2.3.2 Role of model pruning in reducing computational demands

The primary objective of *model pruning* is to identify sub-networks within larger architectures by selectively removing connections. This technique holds considerable appeal for various reasons, particularly for real-time applications on resource-constrained edge devices, which are prevalent in federated learning (FL) and collaborative learning scenarios. Pruning large networks can significantly alleviate the computational demands of inference (Elsen et al., 2020) or hardware tailored to exploit sparsity (Cerebras, 2019; Pool et al., 2021). More recently, the *lottery ticket hypothesis* has emerged (Frankle and Carbin, 2019), suggesting the existence of sub-networks within densely connected networks. These sub-networks, when trained independently from scratch, can attain comparable accuracy to fully trained dense networks (Frankle and Carbin, 2019), revitalizing the field of sparse deep learning (Chen et al., 2020; Renda et al., 2020). This resurgence of interest has also extended into sparse reinforcement learning (RL) (Arnob et al., 2021; Sokar et al., 2021). Pruning techniques in deep learning can broadly be categorized into three groups: methods that induce sparsity before training and during initialization (Lee et al., 2018; Tanaka et al., 2020; Wang et al., 2020; Ohib et al., 2023), during training (Zhu and Gupta, 2018; Ma et al., 2019; Yang et al., 2019; Ohib et al., 2022), and post-training (Han et al., 2015; Frankle et al., 2021). In this work, we leverage findings from methods that induce sparsity at initialization, specifically parameter saliency metrics, to formulate NeuroSFL.

#### 2.3.3 Efficiency in federated learning

For pruning in the FL setting, using a *Lottery Ticket* like approach would result in immense inefficiency in communication. Such methods (Frankle and Carbin, 2019; Bibikar et al., 2022) usually require costly pruning and retraining cycles, often training and pruning multiple times to achieve the desired accuracy vs sparsity trade-off. Relatively few research have leveraged pruning in the FL paradigm (Li A. et al., 2020, 2021; Jiang et al., 2022). In particular, with LotteryFL (Li A. et al., 2020) and PruneFL (Jiang et al., 2022), clients need to send the full model to the server regularly resulting in higher bandwidth usage. Moreover, in Li A. et al. (2020), each client trains a personalized mask to maximize the performance only on the local data. A few recent works (Li A. et al., 2020; Bibikar et al., 2022; Huang T. et al., 2022; Qiu et al., 2022) also attempted to leverage sparse training within the FL setting as well. In particular, Li A. et al. (2020) implemented randomly initialized sparse mask, FedDST (Bibikar et al., 2022) built on the idea of RigL (Evci et al., 2020) which is a prune and re-grow technique, and mostly focussed on magnitude pruning on the server-side resulting in similar constraints and (Ohib et al., 2023) uses sparse gradients to efficiently train in a federated learning setting. In this work, we try to alleviate these limitations which we discuss in the following section.

#### 2.3.4 Privacy in federated learning

Even without sharing raw data, FL can still be vulnerable to privacy attacks such as gradient inversion attacks (Geiping et al., 2020), which can sometimes compromise privacy. Traditional FL algorithms, like federated stochastic gradient descent, are particularly susceptible to these attacks, although methods like Federated Averaging (FedAvg) (McMahan et al., 2017) mitigate this vulnerability to some extent (Geiping et al., 2020; Dimitrov et al., 2022).

Recent research has explored various privacy-preserving techniques in FL. Differential privacy has been proposed to add noise to the model updates to provide strong privacy guarantees (Abadi et al., 2016). Secure aggregation methods ensure that aggregated updates are protected against eavesdropping and manipulation during transmission (Bonawitz et al., 2017). Furthermore, advancements in cryptographic techniques, such as homomorphic encryption and secure multiparty computation, offer promising solutions for preserving privacy in FL settings (Mohassel and Zhang, 2017; Juvekar et al., 2018).

These approaches aim to enhance the robustness of Federated Learning against privacy threats while enabling collaborative model training across distributed data sources. In this work, we primarily focus on improving communication efficiency in FL systems. Although we do not explicitly address privacy, our method can be used in conjunction with other privacy-preservation techniques.

## 3 Method description

In this section we present our proposed method. We first describe the process of discovering a sub-network *f*(**θ**⊙**m**) within the full network *f*(**θ**), where **θ**, the mask**m** ∈ ℝ^*d*^, with ∥**m**∥_0_<*d*. To discover a performant sub-network an importance scoring metric is required, which we describe in Section 3.1.1. Finally, we delineate our proposed method in Section 3.2.

### 3.1 Sub-network discovery

Given a dataset $D={\left\{\left({\text{x}}_{i},y_{i}\right)\right\}}_{i=1}^{n}$ at a site *k*, the training of a neural network *f* parameterized by θ ∈ ℝ^*d*^ can be written as minimizing the following empirical risk as in Equation 3:


(3) $$\begin{array}{l} \underset{\theta }{\mathrm{argmin}}\frac{1}{n}\underset{i}{\sum }L\left(f\left(\theta ;x_{i}\right),y_{i}\right)\;\text{s.t. }\theta \in H \end{array}$$

where ***θ*** ∈ ℝ^*d*^ and L and H are the loss function and the constraint set respectively.

In general, in unconstrained (standard) training the set of possible hypotheses is considered to be $H={\mathbb{R}}^{d}$, where *d* is the model dimension. The objective is to minimize the empirical risk L given a training set ${\left\{\left({\text{x}}_{i},y_{i}\right)\right\}}_{i=1}^{n}\sim D$ at the local client site *k*. Given access to the gradients of the empirical risk on a batch-wise basis, an optimization algorithm such as Stochastic Gradient Descent (SGD) is typically employed to achieve the specified objective. This process generates a series of parameter estimates, ${\left\{\theta_{i}\right\}}_{i=0}^{T}$, where *θ*_0_ represents the initial parameters and *θ*_*T*_ the final optimal parameters. A sub-network within this network is defined as a sparse version of this network with a mask **m** ∈ {0, 1}^|***θ***|^ that results in a masked network *f*(***θ***⊙**m**; ***x***_*i*_). When aiming for a target sparsity level where *k* < *d*, the parameter pruning challenge entails ensuring that the final optimal parameters, *θ*_*T*_, have at most *k* non-zero elements, as denoted by the constraint ||*θ*_*T*_||_0_ ≤ *k*. In many works, this sparsity constraint applies only to the final parameters and not to any intermediate parameter estimates. However, in this work we maintain this sparsity constraint throughout the entire training phase, that is throughout the entire evolution of ***θ*** from ***θ***_0_ to ***θ***_*T*_.

The goal of discovering sub-networks at initialization introduces additional constraints to the previously described framework by requiring that all parameter iterations fall within a predetermined subspace of H. Specifically, the constraints seek to identify an initial set of parameters, *θ*_0_, that has no more than *k*_1_ non-zero elements (∥*θ*_0_∥_0_ ≤ *k*_1_), and ensure that all intermediate parameter sets, *θ*_*i*_, belong to a subspace $\bar{H}\subset H$ for all *i* in {1, …, *T*}, where $\bar{H}$ is the subspace of ℝ^*d*^ spanned by the natural basis vectors {_*e*_*j*_}*j* ∈ supp(*θ*_0_)_. Here, supp(*θ*_0_) represents the support of *θ*_0_, or the set of indices corresponding to its non-zero entries. This approach not only specifies a sub-network at initialization with *k* parameters but also maintains its structure consistently throughout the training.

#### 3.1.1 Connection importance criterion

Lee et al. (2018) introduced a technique for estimating the importance of a connection in a deep learning network inspired by the saliency criterion originally proposed by Mozer and Smolensky (1988). They contributed an important insight, demonstrating that this criterion is remarkably effective in predicting the significance of each connection in a neural network at the initialization phase. The core concept revolves around retaining those parameters that, when altered, would have the most substantial effect on the loss function. This is operationalized by considering a binary vector *c* ∈ {0, 1}^*m*^ and utilizing the Hadamard product ⊙. Consequently, SNIP calculates the sensitivity of connections based on this approach as following:


(4) $$\begin{array}{ll} s\left(\theta ;D\right):= & \frac{\partial L\left(\theta ⊙c\right)}{\partial c}{|}_{c=1}=\frac{\partial L\left(\theta \right)}{\partial \theta }⊙\theta \end{array}$$

After determining *s*(***θ***), the parameters associated with the highest *k* magnitudes of $\left|s{\left(\theta ;D\right)}_{i}\right|$ are retained, where *i* corresponds to the indices of the selected parameters. Essentially, SNIP calculates the importance score of each parameter as its product with the incoming gradient. It prioritizes weights that, regardless of their direction, are distant from the origin and yield large gradient values. It's noteworthy that the objective of SNIP can be reformulated as noted by De Jorge et al. (2020) and Frankle et al. (2021):


(5) $$\begin{array}{l} \underset{c}{\max}S\left(\theta ,c\right):=\underset{i\in \text{supp}\left(c\right)}{\sum }|\theta_{i}\nabla L{\left(\theta \right)}_{i}|\;\text{s.t. }c\in {\left\{0,1\right\}}^{m},||c{||}_{0}=q. \end{array}$$

where *S* is defined to be the saliency scores. It is trivial to note that the optimal solution to the above problem can be obtained by selecting the indices corresponding to the top-*q* values of $s_{i}=|\theta_{i}\nabla L{\left(\theta \right)}_{i}|$.

#### 3.1.2 Iterative connection importance criterion

In this section, we test the effectiveness of iterative-SNIP (De Jorge et al., 2020), which is an iterative version of the application of saliency criterion in Equation 4. We briefly describe the iterative-SNIP next. We assume *q* to be the number of parameters to be preserved post pruning. Given that we have some pruning schedule (similar to learning rate schedule: linear, exponential etc.) to divide *q* into a set of natural numbers ${\left\{k_{t}\right\}}_{t=1}^{T}$ such that *q*_*t*_>*q*_*t*+1_ and *q*_*T*_ = *q*. Now, given the binary masking variable ***c***_*t*_ corresponding to *q*_*t*_, the formulation of pruning from *q*_*t*_ to *q*_*t*+1_ can be made using the connection sensitivity (4) similar to De Jorge et al. (2020) as:


(6) $$c_{t+1}=\;\underset{\hat{\theta },c}{\text{argmax}}\;S(\bar{\theta },\;c)\text{ s.t. }c\in {\left\{0,1\right\}}^{m},\;∥\text{c}{∥}_{0}=k_{q+1},\;c⊙\;c_{t}=\;c,$$

where $\bar{\theta }=\theta ⊙c_{t}$. The constraint ***c***⊙***c***_*t*_ = ***c*** is added to ensure that no previously pruning parameter is re-activated. Assuming that the pruning schedule ensures a smooth transition from one topology to another (∥***c***_*t*_∥_0_ ≈ ∥***c***_*t*+1_∥_0_) such that the *gradient approximation* $\frac{\partial L\left(\bar{\theta }\right)}{\partial \bar{\theta }}{|}_{c_{t}}\approx \frac{\partial L\left(\bar{\theta }\right)}{\partial \bar{\theta }}{|}_{c_{t+1}}$ is valid, Equation 6 can be approximated as solving Equation 5 at $\bar{\theta }$. In the scenario where the schedule parameter is set to *T* = 1, the original SNIP saliency method is recovered. This is basically employing a *gradient approximation* approach between the initial dense network ***c***_0_ = **1** and the resulting mask ***c***. We conduct experiments with IterativeSNIP in the federated neuroimaging setting and present our findings in Section 5.2.

### 3.2 Proposed method

We propose a novel method for *efficient distributed sub-network discovery* for distributed neuroimaging and propose a method for training such sparse models or subnetworks in a communication efficient manner called *Sparse Federated Learning for NeuroImaging* or NeuroSFL with the goal of tackling communication inefficiency during decentralized federated learning with non-IID data distribution in the context of distributed neuroimaging data. The proposed method initiates with the common initialization ***θ***_0_ at all the local client models. Next, importance scores *s*_*j*_ are calculated for each model parameter in the network based on the information from the imaging data available across all the client sites. At this stage, each client has a unique set of importance scores for their parameters in the local network *f* based on the local data available at that site similar to Lee et al. (2018) and De Jorge et al. (2020). As shown in Equation 7, all the clients transmit these scores to each other and a mask **m** is created corresponding to the top-*q* % of the aggregated saliency scores:


(7) $$\begin{array}{l} \text{m}=T_{q}\left(\overset{K-1}{\underset{k=0}{\sum }}s_{k}\right) \end{array}$$

where the *T*_*q*_ is the top-q operator that retains the top *q* percentage of the *s*_*k*_ values by magnitude and sets the rest to zero. This mask is then used to train the model *f*_*k*_(***θ*** ⊙ **m**; ***x***) at site *k* on their local data $\left(x,y\right)\sim D_{k}$.

For the federated training among a total of *K* clients, the clients are trained locally, and at the end of local training they share their trained parameters which are then averaged; we call this a *communication round*. At the start of this local training, each site *k* starts with the same initial model weights ***θ***_0_ which at each site *k* is denoted as *θ*_*k*, 0_ at training step *t* = 0 which are then masked with the generated saliency mask **m** to produce the common masked initialization $\theta_{k,0}^{m}$ as follows:


$$\begin{array}{l} \theta_{k,0}^{m}=\theta_{k,0}⊙\text{m} \end{array}$$

Next these models at each site *k* are trained on their local dataset $\left(x,y\right)\sim D_{k}$.

The masked models *f*(*θ*_*k*, 0_⊙**m**) across all the sites are trained for a total of *T* communication rounds to arrive at the final weights *θ*_*k, T*_ at each local site. In each communication round *t*, only a random subset $F^{\prime }=\left\{f_{1},f_{2},...,f_{K^{\prime }}\right\}$ of *K*′ clients where $F^{\prime }\subseteq F$ the set of all clients, and *K*′ ≤ *K* are trained on their local data. These *K*′ clients are sampled uniformly at random without replacement in a given round but with replacement across different rounds. We sample a subset of clients uniformly instead of including all the clients in a single communication round because previous works have shown that it is computationally more efficient and including more clients in a single round leads to diminishing returns (McMahan et al., 2016). This approach is also a standard practice in the federated learning (FL) literature (Yang et al., 2018; Reddi et al., 2020; Sun et al., 2020; Dai et al., 2022). Since each client has an equal probability of being chosen for participation in a given communication round, over the course of enough communication rounds, all clients will eventually participate. In this work, we train our FL pipeline for a total of *T* = 500 communication rounds, similar to Dai et al. (2022).

At the end of local training on the random subset $F^{\prime }$, the updated weights of the selected clients are aggregated to get the new updated parameters ${\hat{\theta }}_{k,t}^{m}$, which would be the starting weights for the next communication round. When sharing the updated weights only the weights corresponding to the 1's in the binary mask **m** are shared among the clients and with the server, as only these weights are being trained and the rest of the weights are *zero-ed* out. This results in the gains in communication efficiency. To efficiently share the model weights, the clients only share their sparse masked weights $\theta_{F^{\prime }}^{m}=\theta_{F^{\prime }}⊙\text{m}$ among the selected clients in $F^{\prime }$ using the compressed sparse row (CSR) encoding. The algorithm for the training process is delineated in Algorithm 1.

**Algorithm 1:**
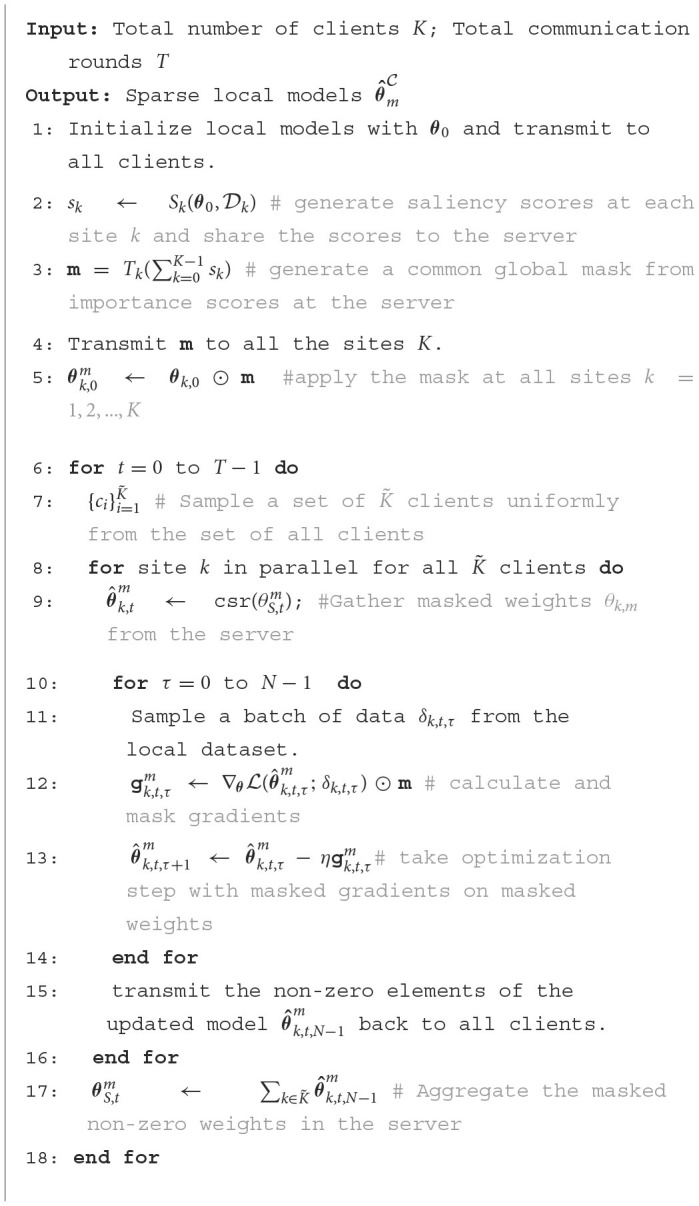
NeuroSFL.

## 4 Experiments

### 4.1 Dataset and non-IID partition

We evaluated NeuroSFL on the ABCD dataset. ABCD study is the largest long-term study of brain development and child health in the US. It recruited over 10 thousand children of 9 and 10 years old from 21 sites and followed them for 10 years with annual behavioral and cognitive assessments and biannual MRI scans (Garavan et al., 2018). Along with multi-session brain MRI scans for structure and function, the ABCD study also includes key demographic information including gender, racial information, socio-economic backgrounds, cognitive development, and mental and physical health assessments of the subjects. The ABCD open-source dataset can be found on the National Institute of Mental Health Data Archive (NDA) (https://nda.nih.gov/). In this study, we used data from the ABCD baseline, which contain 11,875 participants aged 9–10 years.

T1-weighted MRI images were preprocessed using the Statistical Parametric Mapping 12 (SPM12) software toolbox for registration, normalization, and tissue segmentation. Then the gray matter density maps were smoothed by a 6 mm^3^ Gaussian kernel, creating images with the dimensionality of (121, 145, 121) of voxels at Montreal Neuroimaging Institute (MNI) space with each voxel having dimensions of 1.5 × 1.5 × 1.5^3^ mm.

We simulated the heterogeneous data distributions across federated clients through the adoption of two distinct data partitioning strategies. We outline these strategies for generating non-IID data partitions with a comprehensive discussion in Section 4.1.1.

#### 4.1.1 Generating non-IID data partition with Dirichlet distribution

In contrast to centralized data-center training where data batches are often independent and identically distributed (IID), federated learning typically deals with non-IID data distributions across different clients. Hence, to evaluate novel federated learning methods it is crucial to not make the IID assumption to better reflect the real world setting and instead generate non-IID data among clients for evaluation (Hsu et al., 2019). In this section, we discuss the process of generating non-identical data distribution in the client sites using the Dirichlet Distribution, specifically for the context of federated learning.

##### 4.1.1.1 Generating non-IID data from Dirichlet distribution

In this study, we assume that each client independently chooses training samples. These samples are classified into *N* distinct classes, with the distribution of class labels governed by a probability vector ***q***, which is non-negative and whose components sum to 1, that is, *q*_*i*_>0, *i* ∈ [1, *N*] and ∥***q***∥_1_ = 1. For generating a group of non-identical clients, *q*~Dir(α***p***) is drawn from the Dirichlet Distribution, with ***p*** characterizing a prior distribution over the *N* classes and α controls the degree of identicality among the existing clients and is known as the *concentration parameter*.

**Figure 1:**
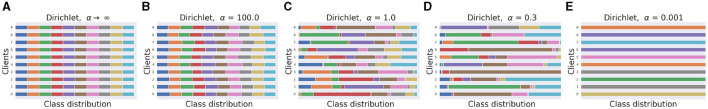
Generating non-identical client data partitions using the Dirichlet Distribution for the Cifar10 dataset among 10 clients. Distribution among classes is represented using different colors. **(A)** Dirichlet, α → ∞ results in identical clients **(B–D)** Client distributions generated from Dirichlet distributions with different concentration parameters α **(E)** Dirichlet, α → 0.0 results in each client being assigned only one particular class.

In this section, we generate a range of client data partitions from the Dirichlet distribution with a range of values for the concentration parameter α for exposition. In Figure 1, we generate a group of 10 balanced clients, each holding an equal number of total samples. Similar to Hsu et al. (2019) the prior distribution ***p*** is assumed to be uniform across all classes. For each client, given a concentration parameter α, we sample a ***q*** from Dir(α) and allocate the corresponding fraction of samples from each client to that client. Figure 1 illustrates the effect of the concentration parameter α on the class distribution drawn from the Dirichlet distribution on different clients, for the CIFAR-10 dataset. When α → ∞, identical class distribution is assigned to each classes. With decreasing α, more non-identicalness is introduced in the class distribution among the client population. At the other extreme with α → 0, each client only consists of one particular class. To create a more realistic FL scenario, we used the value of α = 0.3 for all of our experiments.

### 4.2 Architecture, hyperparameters and experimental details

Here we provide a comprehensive overview of the architecture, hyperparameters, and the experimental setup we use to evaluate our proposed NeuroSFL method on the neuroimaging Adolescent Brain Cognitive Development (ABCD) data. Our study focuses on the task of classifying a participant's sex based on MRI scans, by employing a 3D variant of the well-known AlexNet model (Krizhevsky et al., 2012). The 3D variant was referenced from Abrol et al. (2021), which has a specific channel configuration for the convolutional layers set as: 64C-128C-192C-192C-128C, where “C” denotes channels.

We optimized the learning rate for this task through an exhaustive search ranging from LR = 1 × 10^−3^ to 1 × 10^−6^, achieving a delicate balance between rapid convergence and fine-tuning during training. We employed a batch size of 32 and a learning rate decay factor of 0.998 was applied. We applied varying sparsity levels, ranging from 0%, 50%, 80%, 90%, and 95% to assess the overall performance. A random split of 80/20 was used for training and testing within each individual site. For nonIID setting, as we used alpha = 0.3 for Dirichlet distribution, we enforced an additional constraint of having at least five samples in each client, in order to perform stable training and perform random 80/20 split similar to IID setting. Our training consists of five epochs with 200 communication rounds.

### 4.3 Baselines

We compared our method with both centralized and decentralized baselines. Centralized baseline includes FedAvg (McMahan et al., 2017), FedAvg-FT (Cheng et al., 2021) which are the standard dense baselines, and for the decentralized FL setting, we take the sparse Dis-PFL (Dai et al., 2022).

In FedAvg (McMahan et al., 2017), each client trains its local model using its local data, and then these local models are aggregated or averaged to update the global model. On the other hand, FedAvg-FT (Cheng et al., 2021) extends the FedAvg algorithm by incorporating fine-tuning or transfer learning. Specifically, after the global model is trained using FedAvg, the global model is then fine-tuned or adapted using additional data from a central server or other external sources. This fine-tuning step allows the global model to adapt to new tasks or data distributions beyond what was initially learned from the federated learning process. We also compare with DisPFL (Dai et al., 2022) with varying sparsity levels. DisPFL is a new sparse FL technique that randomly prunes each layer similar to Evci et al. (2020) and uses the prune and regrow method from that work as well, resulting in a dynamically sparse method. The prune and regrow method involves periodically pruning a fraction of the network's weights to zero, and then regrowing new weights in their place, allowing the model to dynamically adjust its sparsity pattern during training.

**Figure 2:**
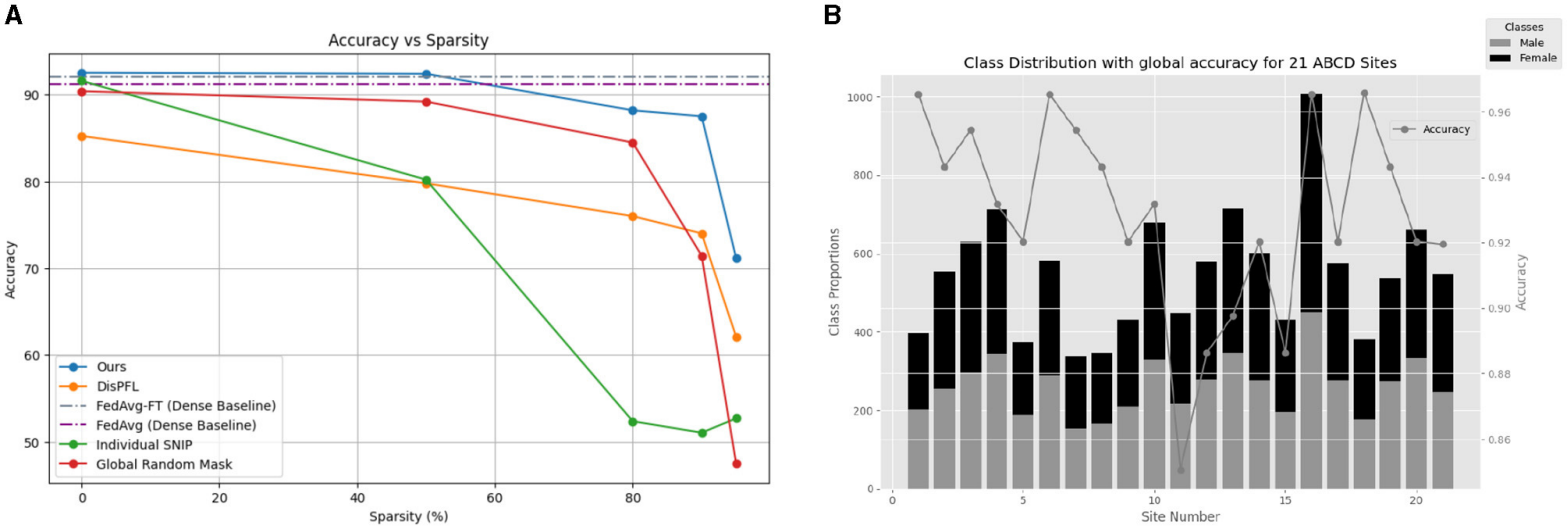
**(A)** Comparison of methods for gender classification using MRI Scans of ABCD dataset. **(B)** Gender differences in each of the 21 ABCD sites along with the performance of the global model on each site with 50% sparsity.

In exploring the impact of using unique local masks instead of a global mask on the performance of FL, we established IndividualSNIP as a baseline, representing an approach where unique local masks are devised from the saliency criterion, and local models are trained based on these masks. Moreover, to isolate the impact of just using *global masking*, that is using the same random mask in all clients, instead of using different unique random masks at different sites we compare our method and competing methods against random global masking as well in Figure 2.

Additionally, with further experiment on how different methods of model pruning and selection impact the performance of our approach, we further experiment with other techniques named IterSNIP and WeightedSNIP. IterSNIP builds upon the traditional SNIP method (Lee et al., 2018) by incorporating multiple minibatches during the training process of mask generation. This approach aggregates saliency scores from these minibatches to generate a comprehensive and robust pruning mask. Conversely, WeightedSNIP adopts a different strategy, deriving a global mask through a weighted average of saliency scores based on the frequency of data at each site, and assigning importance levels to individual sites based on the amount of data at sites.

### 4.4 Experiments in real-world FL system

In order to demonstrate the use-case of the NeuroSFL in real world scenario, we further aim to perform extensive experiments in a real-world FL system by making use of Coinstac (Plis et al., 2016), a cutting-edge open-source federated learning solution designed for collaborative neuroimaging endeavors at scale. Deployed in real-world scenarios, Coinstac embodies a paradigm shift in collaborative research, transcending traditional boundaries and fostering synergistic interactions among researchers worldwide. Coinstac's architecture facilitates decentralized computations across a distributed network of geographically dispersed client nodes, seamlessly integrating diverse computational tasks while safeguarding data privacy through state-of-the-art differential privacy mechanisms. Having said this, our experiment leverages Coinstac's robust infrastructure to benchmark our method against the standard dense FedAvg algorithm (McMahan et al., 2017) within a practical real-world context. Our evaluation spans five diverse client locations, spanning from North Virginia to Frankfurt, each representing a distinct geographical node within Coinstac's decentralized network. By meticulously assessing the mean communication timereflecting the duration for the server model to aggregate all client weights during each communication round, we demonstrate the efficiency of our algorithm in optimizing federated learning workflows. Our investigation encompasses five local client models, each featuring varying sizes or depths of ResNet architectures while maintaining a sparsity level of 90% across experiments.

## 5 Results and discussion

### 5.1 Effect of varying sparsity levels

We first explore the effect of sparsity on IID data in Section 5.1.1 and then explore the efficacy of NeuroSFL on non-IID data in Section 5.1.2.

#### 5.1.1 Effect of varying sparsity levels on IID data

The performance of various methods across different sparsity levels was evaluated, as presented in Table 1, and visually presented in Figure 2. Sparse baselines, including Ours (*NeuroSFL*), IndividualSNIP, DisPFL (Dai et al., 2022), and Global Random Mask, were compared against dense baselines such as FedAvg-FT (Cheng et al., 2021) and FedAvg (McMahan et al., 2017). Notably, our proposed *NeuroSFL*, exhibited robust performance across varying sparsity levels, achieving an accuracy of 92.52% at 0% sparsity and maintaining high accuracy even at higher sparsity levels, with 71.18% accuracy at 95% sparsity. In comparison, IndividualSNIP demonstrated decreasing accuracy as sparsity increased, with a significant drop to 52.70% at 95% sparsity. This is in line with expectation as individual-SNIP only incorporates the saliency scores from a single site at random and does not incorporate information from the datasets at all the participating cites.

**Table 1:** Performance comparison of different methods and sparsity levels.

**Method**	**Sparsity (%)**				
	**0%**	**50%**	**80%**	**90%**	**95%**
**Sparse baselines**					
Ours (NeuroSFL)	92.52%	92.4%	88.19%	87.5%	71.18%
DisPFL	85.24%	79.78%	76.00%	74.01%	62.12%
IndividualSNIP	91.59%	80.20%	52.37%	51.04%	52.70%
Global Random Mask	90.39%	89.20%	84.48%	71.44%	47.53%
**Dense baselines**					
FedAvg-FT	92.1% (dense baseline)				
FedAvg	90.5% (dense baseline)				

Sparse baselines include Ours- (NeuroSFL), DistPFL, IndividualSNIP, and Global Random Mask. Dense baselines include FedAvg and FedAvg-FT.

Moreover, in contrast to NeuroSFL, DisPFL, and Global Random Mask also showcased diminishing accuracy with increasing sparsity, highlighting the effectiveness of our proposed approach in mitigating the adverse effects of sparsity on model performance on neuroimaging data. Notably, Global Random Mask outperformed DisPFL on lower sparsities, suggesting that in general global random masks might be more suitable for federated applications compared to even targeted unique local masks which DisPFL employs.

Dense baselines, such as FedAvg-FT and FedAvg, even while being *not sparse* and using full communication achieved comparable performances to NeuroSFL in the non-extreme sparsity region. NeuroSFL even surpassed the performance of dense baselines at a sparsity level of 50%, highlighting the effectiveness of our proposed sparse method in optimizing model performance while reducing communication costs. Furthermore, Figure 2B illustrates that the single global model trained with *NeuroSFL* demonstrated excellent performance for data within each site, emphasizing the model's effectiveness in capturing site-specific characteristics while maintaining high accuracy.

**Figure 3:**
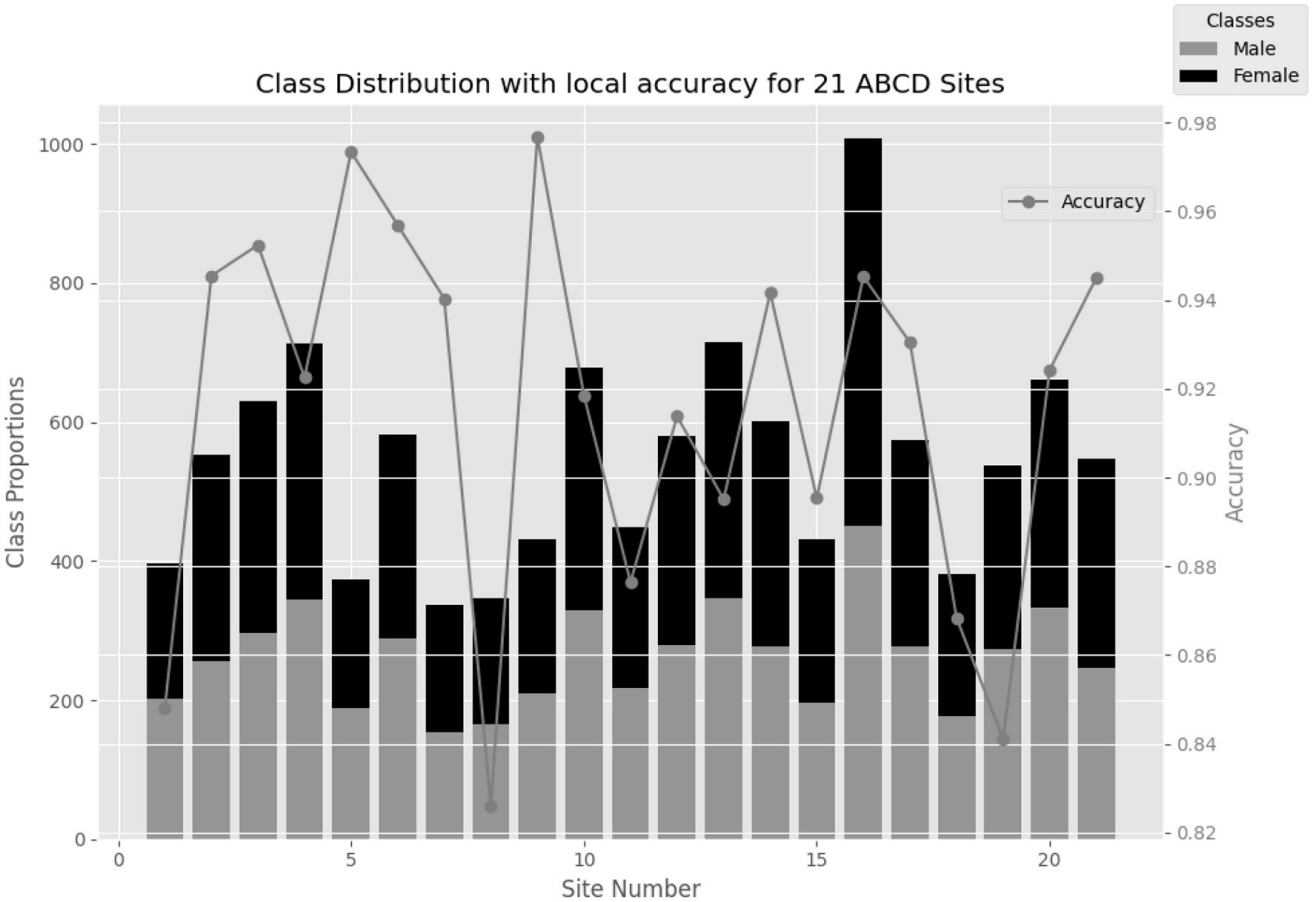
Gender differences in each of the 21 ABCD sites along with the performance of the local models on each site with 50% sparsity.

Additionally, in Figure 3, it is observed that the performance of local models trained with *NeuroSFL* remains consistently robust across non-IID states of local data, indicating the model's versatility and reliability in various data distribution scenarios.

#### 5.1.2 Effect of varying sparsity levels on non-IID data

In this section, we explore the influence of changing sparsity levels on our model's performance on non-IID data across different client configurations: 10, 20, and 30 clients with f1-score as a metric. For all configurations, we employ the Dirichlet Distribution with alpha = 0.3 across various sparsity levels.

##### 5.1.2.1 10 Clients

**Figure 4:**
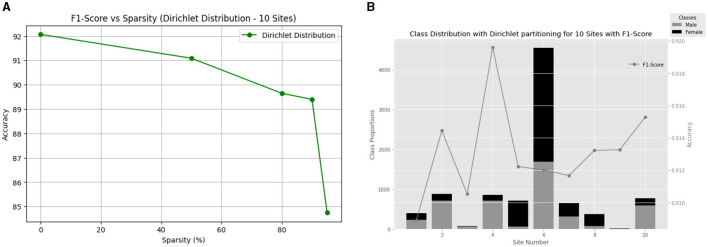
**(A)** Comparison of methods for gender classification using non-IID Dirichlet distribution with alpha = 0.3 and varying sparsity levels. **(B)** Gender differences in each of the 10 ABCD sites along with the performance of the model.

We begin by examining the model's performance with 10 clients. Figure 4 provides a visual representation of the F1-score versus sparsity relationship, showcasing the consistent performance achieved across different sparsity levels (Figure 4A). Additionally, Figure 4B illustrates the class distribution with Dir(0.3) for the ABCD dataset for 10 clients and their final local test F1-score. The Dirichlet partition results in an uneven data distribution, as visually confirmed by Figure 4B.

Notably, our model demonstrated robust performance across different sparsity levels, ranging from 84.75 to 92.07%. These results underscore the resilience of our approach in maintaining high performance even under significant sparsity constraints. This resilience suggests that our model's effectiveness extends beyond homogeneous datasets, making it suitable for deployment in federated learning scenarios with diverse client characteristics.

##### 5.1.2.2 20 Clients

**Figure 5:**
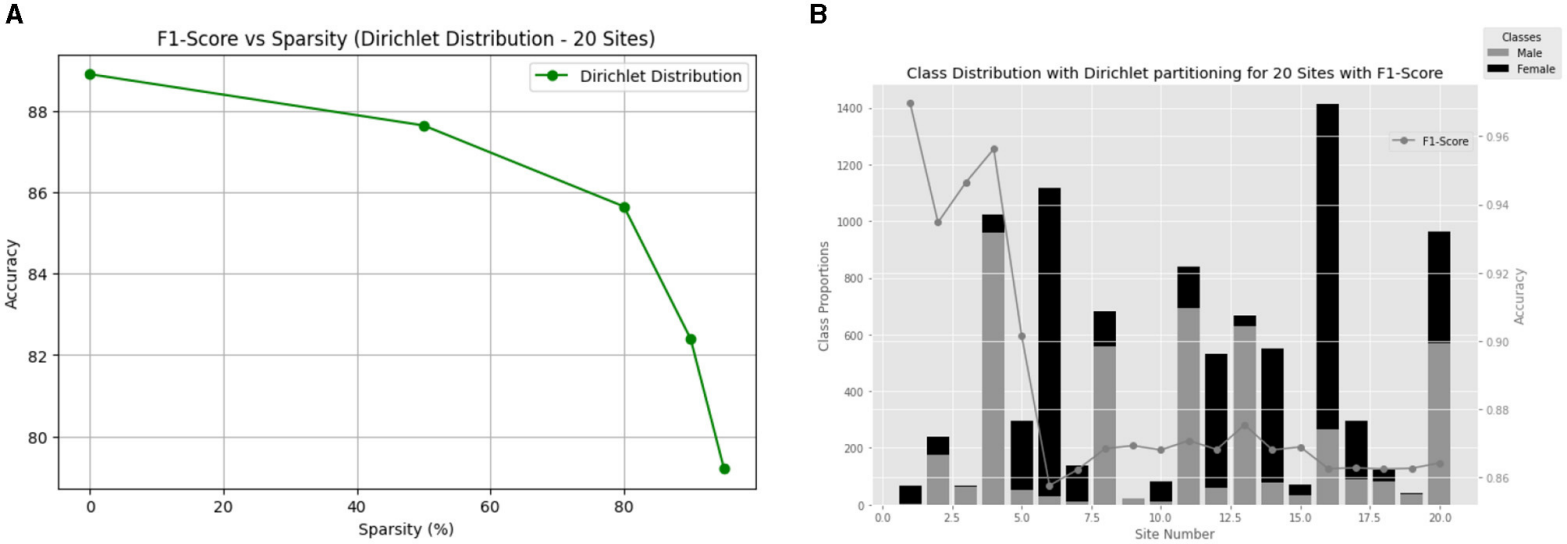
**(A)** Comparison of methods for gender classification using non-IID Dirichlet distribution with alpha = 0.3 and varying sparsity levels. **(B)** Gender differences in each of the 20 ABCD sites along with the performance of the model.

Expanding our analysis to 20 clients, we investigate how varying sparsity levels impact our model's performance on non-IID data. Figure 5 provides a visual representation of the F1-score versus sparsity relationship, highlighting the consistent performance achieved across different sparsity levels (Figure 5A). Additionally, Figure 5B illustrates the class distribution with Dir(0.3) for the ABCD dataset for 20 clients and their final local test F1-score.

The findings indicate a similar pattern to the 10-client case, with the model maintaining notable F1-scores across varying sparsity levels, with the lowest performance being 79.21% under the highly sparse constraint of 95% sparsity. This highlights the model's ability to generalize effectively even with significant data sparsity.

##### 5.1.2.3 30 Clients

**Figure 6:**
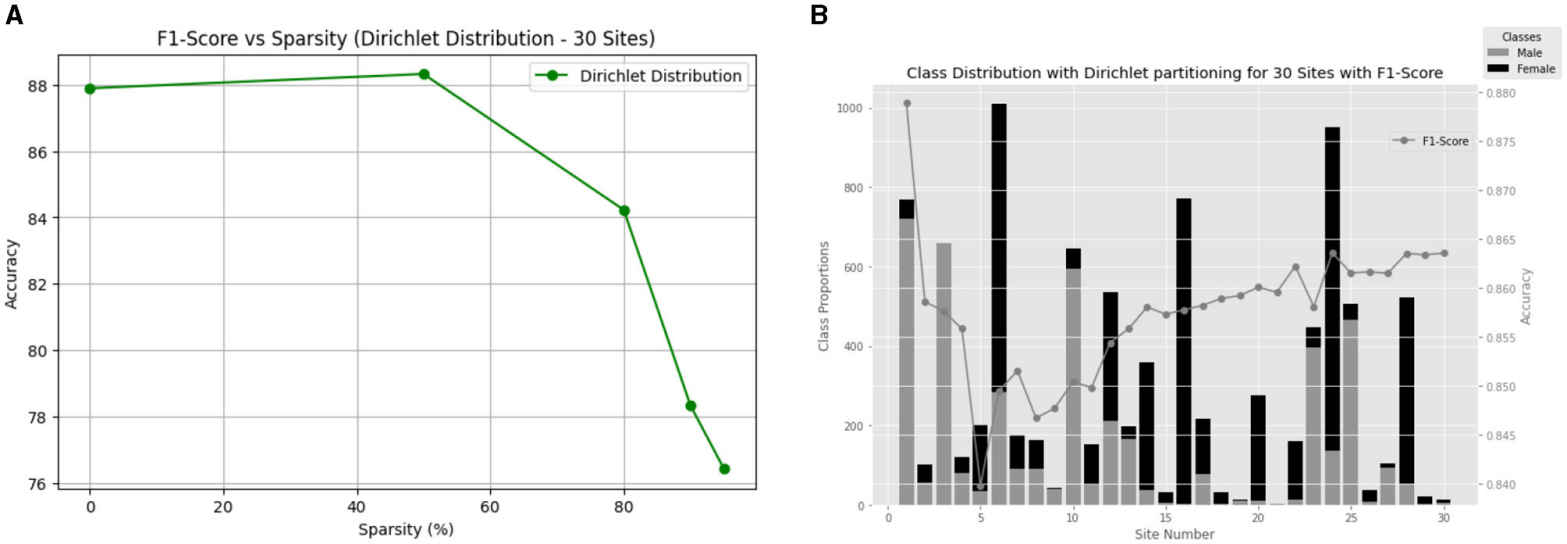
**(A)** Comparison of methods for gender classification using non-IID Dirichlet distribution with alpha = 0.3 and varying sparsity levels. **(B)** Gender differences in each of the 30 ABCD sites along with the performance of the model for 50% sparsity.

Finally, we extend our analysis to 30 clients to further understand the impact of varying sparsity levels on non-IID data with larger number of clients. Figure 6 provides a visual representation of the F1-score versus sparsity relationship, showcasing the consistent performance achieved across different sparsity levels (Figure 6A). Additionally, Figure 6B illustrates the class distribution with Dir(0.3) for the ABCD dataset for 30 clients and their final local test F1-score.

The results reveal a similar trend to the 20-client scenario, with the model achieving notable F1-scores across varying sparsity levels. However, for the highly sparse condition of 95%, there is a slight drop in F1-score to 76.42%. This decline can be attributed to the increased difficulty for the model to generalize with such sparse data under the additional restriction of having larger clients. Despite this challenge, our model maintains its effectiveness across a diverse range of sparsity levels, indicating its potential for practical applications in federated learning scenarios with a larger number of client sites.

### 5.2 IterativeSNIP performance

We evaluate the performance of IterSNIP and WeightedSNIP to explore their efficacy in sparse FL scenarios. Table 2 summarizes the accuracy results obtained at 50% sparsity for different iterations of IterSNIP and WeightedSNIP. IterSNIP, with varying numbers of iterations (1, 10, and 20), demonstrated consistent performance with increasing iterations, achieving accuracies of 92.4%, 91.82%, and 92.67%, respectively. These results suggest that utilizing multiple iterations to obtain SNIP masks does not necessarily enhance model performance in scenarios with sparsity constraints, and especially when used on neuroimaging data. This is a departure from the single-node case on natural image datasets such as CIFAR10 or CIFAR100 (De Jorge et al., 2020). Similarly, WeightedSNIP, which incorporates weighted averages of saliency scores, achieved an accuracy of 92.10% and does not outperform the vanilla averaging technique. This proves that our model is robust enough to find a sparse mask, with minimal effect from the amount of data at each sites.

**Table 2:** Performance comparison of IterSNIP with different iterations and WeightedSNIP in terms of accuracy at sparsity of 50%.

**Method**	**Iterations**	**Accuracy (50% sparsity)**
IterSNIP	1 Iteration	92.40%
	10 Iteration	91.82%
		20 Iteration	92.67%
WeightedSNIP	1 Iteration	92.10%

### 5.3 Wall-time efficiency gains in the real world COINSTAC system

The results of the ensuing comparative analysis as delineated in Table 3, demonstrate the tangible speed enhancements achieved by our proposed methodology NeuroSFL as compared to the standard FedAvg in a real-world setting. Importantly, our results indicate that our approach consistently outperforms FedAvg across all ResNet architectures. For instance, in the case of ResNet32, our method achieves a communication time of 0.238 ± 0.02 s, compared to 0.285 ± 0.04 s for FedAvg, resulting in a speedup of 1.20*times*. This trend continues across deeper architectures, with our technique demonstrating significant improvements in communication efficiency. For instance, for ResNet110, our method achieves a remarkable speedup of 2.32x over FedAvg, showcasing its ability to handle complex models with greater efficiency. These empirical findings underscore the importance of sparse federated techniques like NeuroSFL, thereby propelling collaborative neuroimaging research to unprecedented heights.

**Table 3:** Comparison of communication time between FedAvg and NeuroSFL on Cifar10 for ResNet architectures of different depth.

**Architecture**	**Accuracy**	**Communication time (s)**		**Speed up**
		**FedAvg**	**NeuroSFL**	
ResNet32	90.52%	0.285 ± 0.04	0.238 ± 0.02	1.20 *times*
ResNet44	89.65%	0.409 ± 0.06	0.328 ± 0.04	1.24 *times*
ResNet56	93.74%	0.531 ± 0.07	0.407 ± 0.06	1.30 *times*
ResNet110	93.25%	1.812 ± 0.33	0.781 ± 0.13	2.32 *times*

### 5.4 Sparsity vs. accuracy performance comparison

In this section we analyze and interpret the results from Section 5. First, we probe the reasons behind the performance gains in comparison to a state of the art federated sparse learning method (Dai et al., 2022).

In a specific comparison with DistPFL, we can see that NeuroSFL consistently performs better than DisPFL in a range of sparsities in the selected tasks. This is probably due to a better choice of the initial sparse sub-network using the importance criterion. Another difference is that, in DisPFL different local clients have different levels of sparsity and a final model averaging is done, where the final model becomes denser due to the union of many sparse subnetworks. We however retain the same mask in all the clients and start from the same initialization in all the clients, result in equivalent sparsity in all the clients; this also leaves open the potential of keeping sparse global models in a centralized FL setting.

## 6 Conclusion and future work

In this work, we propose and analyze a novel communication-efficient FL method for neuroimaging called NeuroSFL. By extending a gradient-based parameter importance criterion to the FL setting, we achieve reduced communication costs and better bandwidth in decentralized training. Our method leverages the nature of local data distribution, resulting in a client data-aware global sparse mask. This leads to savings in communication time and bandwidth during sparse training. We tested our approach on the ABCD dataset and reported improved performance compared to contemporary methods. Overall, our sparse FL technique enhances communication time, making it suitable for bandwidth-limited settings without compromising accuracy.

However, more exploration is needed regarding privacy considerations and performance in more complex tasks. Although FL models inherently provide more privacy compared to other training pipelines, such as training with centralized data (Li Q. et al., 2021), they can still be susceptible to more sophisticated forms of attacks (Geiping et al., 2020). Sparse gradients can often result in more privacy-preserving methods (Zhang et al., 2023), hence it is likely our method would enjoy similar advantages. Moreover, our method should be easily extensible to incorporate differential privacy techniques (Ouadrhiri and Abdelhadi, 2022). We leave such explorations for future work.

## Data Availability

Publicly available datasets were analyzed in this study. This data can be found here: https://nda.nih.gov/.
